# Efficacy and safety of carfilzomib in relapsed and/or refractory multiple myeloma: systematic review and meta-analysis of 14 trials

**DOI:** 10.18632/oncotarget.25281

**Published:** 2018-05-04

**Authors:** Chintan Shah, Rohit Bishnoi, Yu Wang, Fei Zou, Harini Bejjanki, Samip Master, Jan S. Moreb

**Affiliations:** ^1^ Division of Hospital Medicine, University of Florida, Gainesville, Florida, USA; ^2^ Department of Biostatistics University of Florida, Gainesville, Florida, USA; ^3^ Division of Hematology/Oncology, Louisiana State University, Shreveport, Louisiana, USA; ^4^ Division of Hematology/Oncology, University of Florida, Gainesville, Florida, USA

**Keywords:** carfilzomib, kyprolis, multiple myeloma, response, efficacy

## Abstract

**Objective:**

Carfilzomib (Carf) is a second-generation proteasome inhibitor approved for patients with relapsed and/or refractory multiple myeloma (RRMM) who failed ≥ 1 prior lines of therapy. We performed a systematic review of Carf literature with meta-analysis to determine the efficacy and safety in RRMM patients.

**Methods:**

Based on literature search, we included a total of 14 eligible phase I/II, phase II and phase III Carf based clinical trials. The cumulative incidence and odds ratios (OR) were calculated with random effect model, using ‘’R’’ software with metaphor package.

**Results:**

2906 evaluable RRMM patients from published clinical trials included. The pooled overall response rate (ORR) was 45% (95% CI: 29–62). The pooled clinical benefit rate (CBR) was 56% (95% CI: 41–71). OR from 3 randomized clinical trials showed that Carf significantly improved ORR and CBR compared to control groups (OR 2.4, *P* < 0.0001; 2.02, *P* = 0.0007, respectively). Subgroup analysis showed significantly better ORR (*P* < 0.0001) and CBR (*P* < 0.001) with combination regimens compared to monotherapy. Response was significantly higher with high dose of Carf (>20/27 mg/m^2^) compared to standard dose (ORR 65% vs. 35%, *P* = 0.03). Compared to control group, the OR of developing cardiotoxicity (*P* = 0.002) and hypertension (*P* < 0.0001) were significantly higher with Carf, while no difference in peripheral neuropathy (*P* = 0.28).

**Conclusions:**

Carf produces significantly better responses with acceptable safety profile in RRMM patients. Combination regimens and higher dose Carf offers better response with no significant extra toxicity. Its efficacy is regardless of cytogenetics or disease stage. Incidences of cardiotoxicity and hypertension seem higher with Carf.

## INTRODUCTION

Survival rates have improved in multiple myeloma (MM) patients since the approval of the novel therapeutics such as proteasome inhibitors (PI) and immunomodulators (IMiDs) [1]. Bortezomib (Bort) is the first-in-class PI, which is approved in the USA for the treatment of patients with MM and mantle cell lymphoma [2]. It is modified dipeptidyl boronic acid which reversibly inhibits the protease activity of the 26S proteasome responsible for degradation of intracellular proteins through the ubiquitin-proteasome pathway [3]. Inhibition of proteasomal activity disrupts the cell signaling pathways which lead to apoptosis [4]. Carfilzomib (Carf) is the second-generation PI that irreversibly inhibits 20S proteasome, and is approved as a combination therapy with dexamethasone (Dexa) or with lenalidomide (Len) plus Dexa for the treatment of patients with relapsed and/or refractory MM (RRMM) who have received one to three prior lines of therapy and as a single agent for patients with RRMM who have received one or more lines of therapy [5].

In spite of all these advances, MM still imposes a major therapeutic challenge as the majorities of the patients eventually develop resistance to these agents and relapse [6]. MM has remained incurable disease as tumor typically recurs more aggressively after each relapse and ultimately treatment-refractory disease develops and leads to the demise of patients [7]. There is no standard uniform treatment for RRMM [8]. Various randomized and non-randomized clinical trials have used Carf either as a single agent or in various combinations with other agents, and with variable dosing schedules which have resulted in a wide range of response rates. Response rates and treatment choices depend on various disease and patient-related factors. However specific toxicity profile can impact treatment selection, especially in this group of RRMM patients as they are usually heavily pre-treated. Current standard dosing schedule of Carf is 20 mg/m^2^/day in cycle 1 and if tolerated increase the dose in subsequent cycles to 27 mg/m^2^/day [5]. Many clinical trials have used higher doses and slower infusion rate.

Other preclinical, as well as clinical studies suggest that Carf based combination regimens can provide synergistically superior response rates, but the impact on progression-free survival (PFS) and overall survival (OS) remains unclear [9–11]. Given all the published information about the experiences with Carf, we feel that clarification of its effectiveness as a single agent or in combination is very much needed. Here we present systematic review and meta-analysis of published clinical trials on Carf in patients with RRMM. We analyzed efficacy of Carf in RRMM patients and performed various subgroup analyses to understand effects of different doses of Carf (high vs. standard) and regimens (monotherapy vs. combination) into response rates as well as adverse events. We also performed subgroup analyses to evaluate efficacy of Carf in high risk cytogenetics and different ISS stages. Furthermore, we analyzed commonly reported adverse events including cardiotoxicity with respect to different doses of Carf.

## RESULTS

Based on our search criteria, we identified a total of 14 clinical trials [12–25] which used Carf based regimens in RRMM patients with a total of 2938 enrolled patients and 2906 evaluable patients. Thirty-two patients were excluded from analysis due to various reasons such as incorrect enrollment (2), missing baseline and/or post-baseline disease assessment (12), intolerance to maximum dose criteria of the study (12), self-withdrawal (1), reason not mentioned (5). The median age of the patients ranged between 61.5–68.5 years. Characteristics of patients with the response and long-term outcomes from different studies are summarized in Tables 1–3. There were three randomized controlled trials (RCTs) with 2036 enrolled patients, 1017 in Carf group and 1019 in control group [16, 17, 21]. A total of 7 clinical trials used Carf in combination with other agents, such as Dexa in four studies [12, 15, 16, 25], Len and Dexa in two studies [21, 24] and panobinostat in one study [13] as shown in Tables 1–3.

**Table 1:** Patient characteristics, response and long-term outcomes summary from phase III studies with control groups

Author, Year	Regimen used	Carf dosing (mg/m^2^)	Median age (years)	Patients analyzed, n	CR, *n* (%)	VGPR, n (%)	ORR, *n* (%)	CBR, *n* (%)	Median DOR (mos)	Median PFS (mos)	Median OS (mos)	Type of cardiac events
Dimopoulos MA *et al*., 2016	Carf, Dexa	20 (Days 1, 2 of cycle 1) f/b 56	65	464	58 (13)	194 (42)	356 (77)	380 (82)	NA	18.7	47.6	Cardiac failure, Ischemic heart disease
(ENDEAVOR)	Bort, Dexa		65	465	29 (6)	104 (22)	290 (62)	343 (74)	NA	9.4	24.3	
Hajek R *et al*., 2017 (FOCUS)	Carf	20 (Days 1, 2 of cycle 1) f/b 27	63	157	1 (1)	5 (3)	30 (19)	49 (31)	7.2	3.7	10.2	Cardiac failure
	Pred or Dexa		66	158	0 (0)	5 (3)	18 (11)	33 (21)	9.5	3.3	10	
Stewart AK *et al*., 2015 (ASPIRE)	Carf, Len, Dexa	20 (Days 1, 2 of cycle 1) f/b 27	64	396	126 (31.8)	277 (69.9)	344 (87.1)	359 (91)	28.6	26.3	NA	cardiac failure, ischemic heart disease
	Len, Dexa		65	396	37 (9.3)	160 (40.4)	264 (66.7)	302 (76.3)	21.2	17.6	NA	

Abbreviations: Carf, carfilzomib; CR, complete response; VGPR, very good partial response; ORR, overall response rate; CBR, clinical benefit rate; PFS, progression free survival; OS, overall survival, Pred, prednisone; Dexa, dexamethasone; Len, lenalidomide; Bort, bortezomib; NA, not available; f/b, followed by; mos, months; MI, myocardial infarction; CHF, congestive heart failure; CAD, coronary artery disease;

**Table 2:** Patient characteristics, response and long-term outcomes summary from phase II studies

Author, Year	Regimen used	Carf dosing (mg/m2)	Median age (years)	Pts analyzed, n	CR, n (%)	VGPR, n (%)	ORR, n (%)	CBR, n (%)	Median DOR (mos)	Median PFS (mos)	Median OS (mos)	Type of cardiac events
Lendvai N *et al*., 2014	Carf	20 (Days 1, 2 of cycle 1) f/b 56	63	42	1 (2)	9 (21)	23 (55)	25 (60)	11.7	4.1	20.3	Heart failure
Siegel DS *et al*., 2012	Carf	20 (Days 1, 2 of cycle 1) f/b 27	63	257	1 (0.4)	13 (5.1)	61 (23.7)	95 (37)	7.8	3.7	15.6	Cardiac failure, cardiac arrest, MI
Jagannath S *et al*., 2012	Carf	20 (Days 1, 2, 8, 9, 15, 16)	63.5	46	NA	NA	7 (16.7)	10 (24)	7.2	3.5	NA	Cardiac failure
Wang M *et al*., 2013	Carf, Len, Dexa	20 (Days 1, 2 of cycle 1) f/b 27	61.5	84	1 (1.2)	30 (35.7)	58 (69.0)	64 (76)	18.8	11.8	NA	MI, sick-sinus syndrome, CAD
Vij R *et al*., 2012	Carf	20 (Days 1, 2 of cycle 1) f/b 27	63	35	1 (2.9)	1 (2.9)	6 (17.1)	11 (31.4)	NA	4.6	29.9	CHF
Vij R *et al*.,2012	Carf	20 (Days 1, 2 of cycle 1) f/b 27	66	126	3 (2.4)	26 (20.6)	60 (47.6)	78 (62)	NA	NA	NA	CHF
Badros AZ *et al*., 2013	Carf, Dexa	15 (cycle 1) f/b 20 (cycle 2) f/b 27	64	47	0	0	12 (25.5)	15 (32)	7.9	NA	NA	CHF

Abbreviations: See Table 1.

**Table 3:** Patient characteristics, response, and long-term outcomes summary from phase I/II trials

Author, Year	Regimen used	Carf dosing (mg/m^2^)	Median age (years)	Pts analyzed, *n*	CR, *n* (%)	VGPR, *n* (%)	ORR, *n* (%)	CBR, *n* (%)	MedianDOR (mos)	Median PFS (mos)	Median OS (mos)	Type of cardiac events
Watanabe T *et al*., 2016	Carf, Dexa	20 (Days 1, 2 of cycle 1) f/b 27	67	50	0	2 (4)	10 (20)	14 (28)	9.5	5.1	23.4	CHF, atrioventricular block, cardiomyopathy
Berenson JR *et al*., 2016	Carf, Dexa	20 (Days 1 of cycle 1) f/b 45 or 56 or 70 or 88 (once weekly)	68.5	104	11 (11)	34 (33)	77 (77)	84 (84)	NA	12.6	NA	MI, atrial fibrillation, cardiorespiratory arrest, CHF
Berdeja JG *et al*., 2015	Carf, Pano	20 (Days 1, 2 of cycle 1) f/b 27 or 36 or 45 ^€^	66	42	NA	14 (33)	28 (67%)	33 (79)	11.6	7.7	NA	CHF
Berenson JR *et al*., 2014	Carf^#^	20 (cycle 1) f/b 27 (cycle 2) f/b 36 (cycle 3) f/b 45 (cycle 4)^*^	67	37	3 (8.1)	6 (16.2)	16 (43.2)	23 (62.2)	9.9	8.3	15.8	Tachyarrhythmia, CHF

Abbreviations: See Table 1

^#^in various combinations with immunomodulatory drug (Thal or Len), pegylated liposomal doxorubicin, glucocorticoids, cyclophosphamide, methylprednisone, bendamustine

^€^4 (9.5%) out 42 patients had maximum carfilzomib dose ≤ 27 mg/m^2.^

^*^10 (27%) out 37 patients had maximum carfilzomib dose ≤ 27 mg/m^2.^

### Response rates and survival outcomes

**Figure 1:**
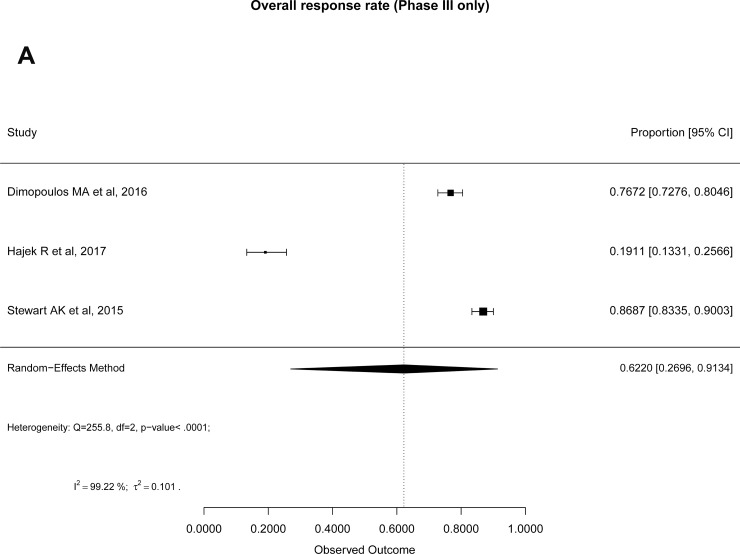

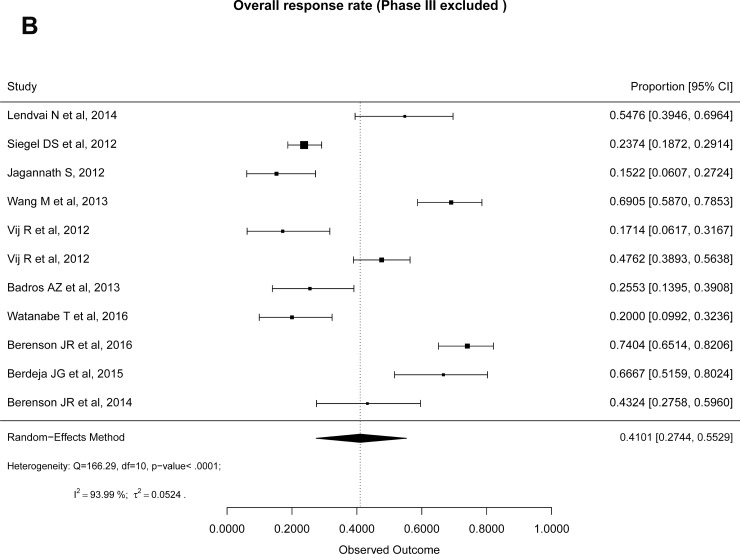

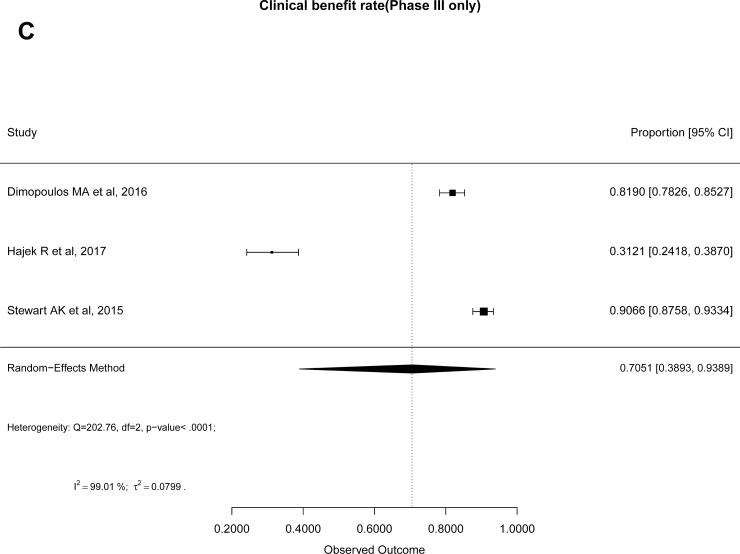

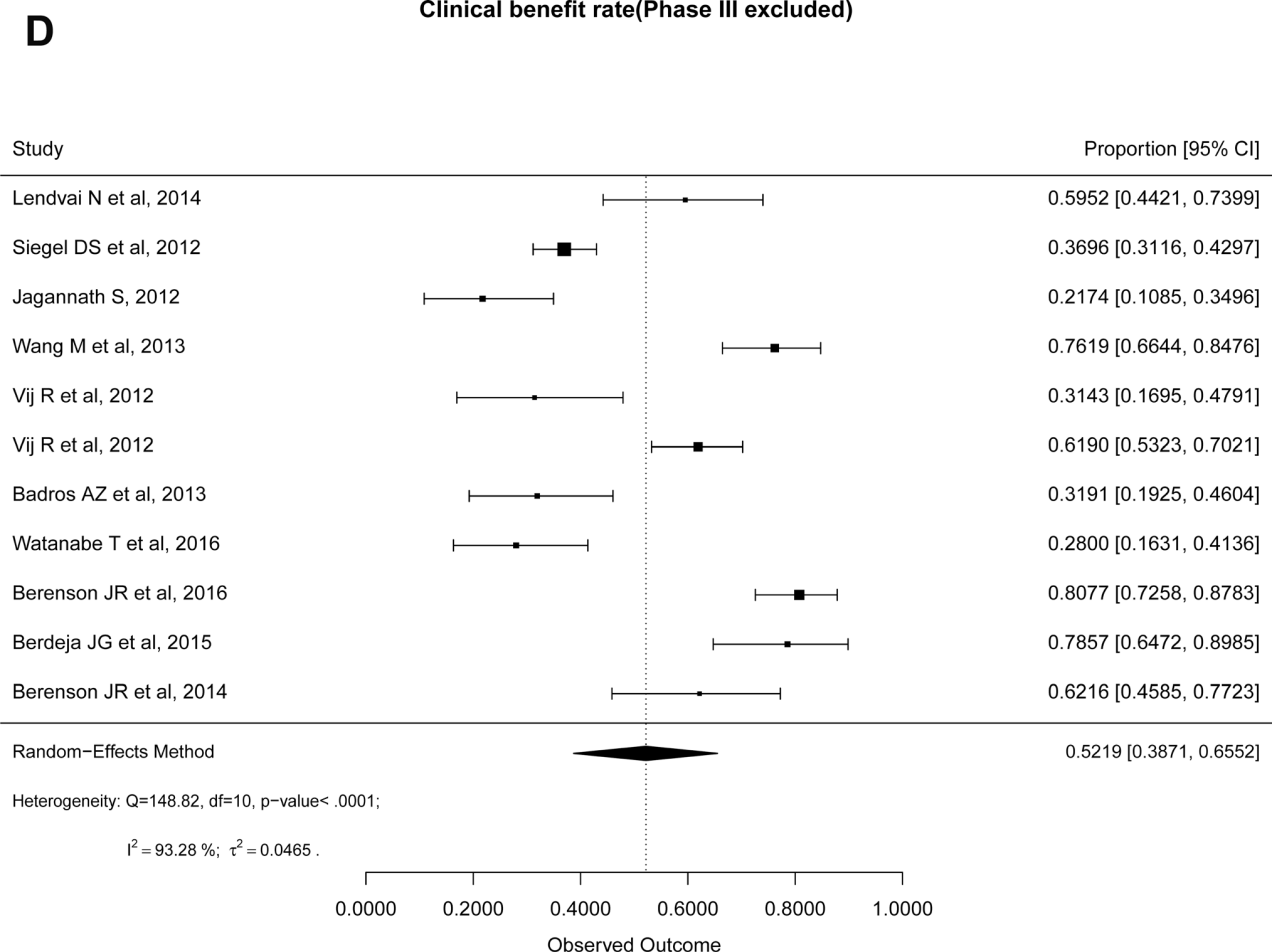
Forest plots of pooled response rates in Carf treated RRMM patients: (**A**) Overall response rate of phase III studies, (**B**) Overall response rate of phase II and I/II studies, (**C**) Clinical benefit rate of phase III studies, (**D**) Clinical benefit rate of phase II and I/II studies.

The pooled overall response rate (ORR) (CR+VGPR+PR) from all 14 included studies was 45% (95% CI: 29–62) by random effect model. Similarly, the overall clinical benefit rate (CBR) (ORR+MR) was 56% (95% CI: 41–70). High heterogeneity between studies (I^2^~97) was observed for both. Therefore, we report separate analysis for phase III studies as compared to the rest. The pooled ORR for phase III studies was 62% (95% CI: 26–91) and rest of the studies was 41% (95% CI: 27–55) by random effect model (Figure 1A, 1B). Similarly, the pooled CBR from phase III studies was 70% (95% CI: 38–93), while it was 52% (95% CI: 38–65) for the other phase I/II studies (Figure 1C, 1D). There was no evident publication bias found as funnel plot remained symmetrical. Table 4 shows overall proportions for complete response (CR) and very good partial response (VGPR) and their respective I^2^ for heterogeneity between studies.

**Figure 2:**
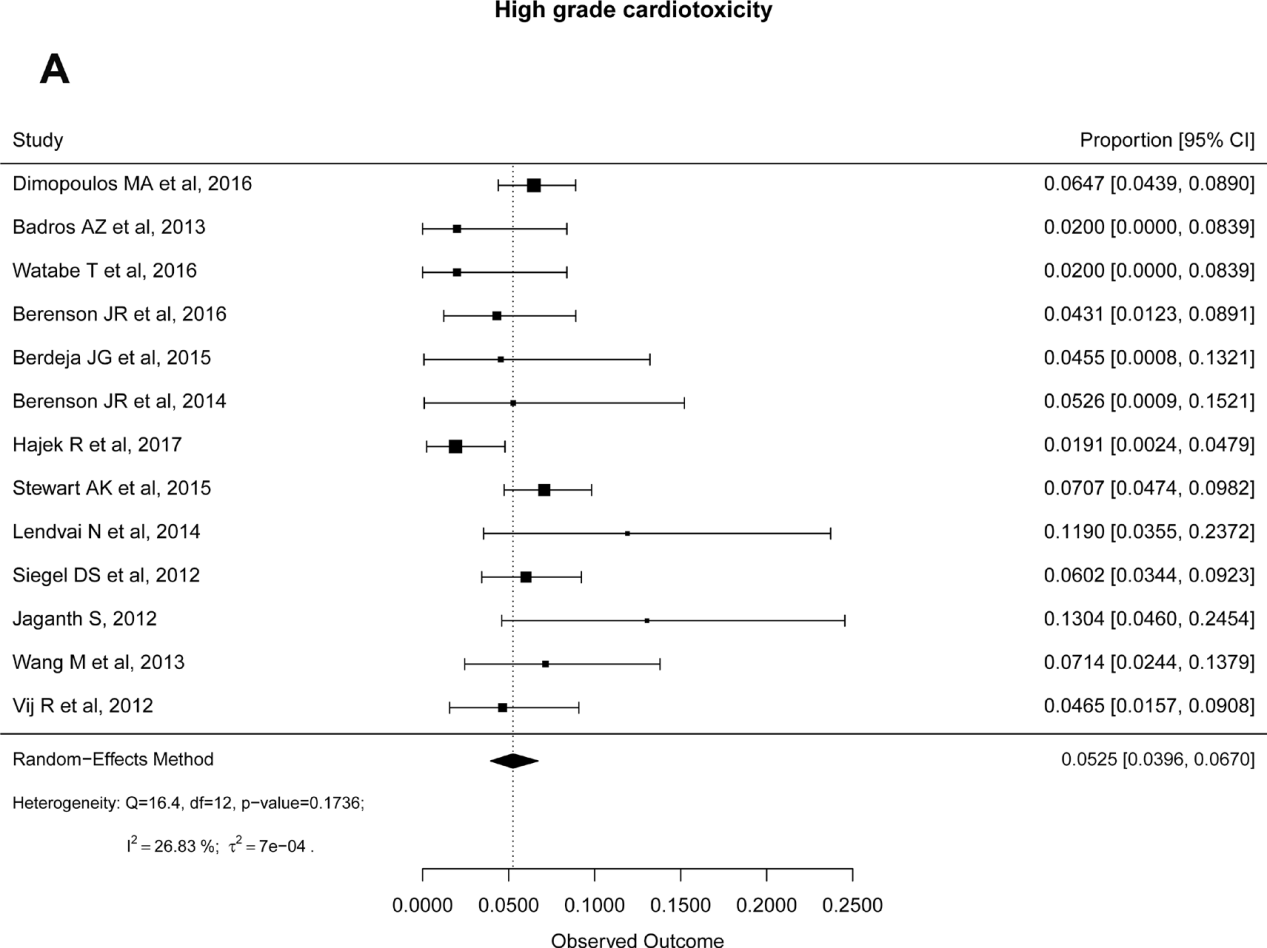

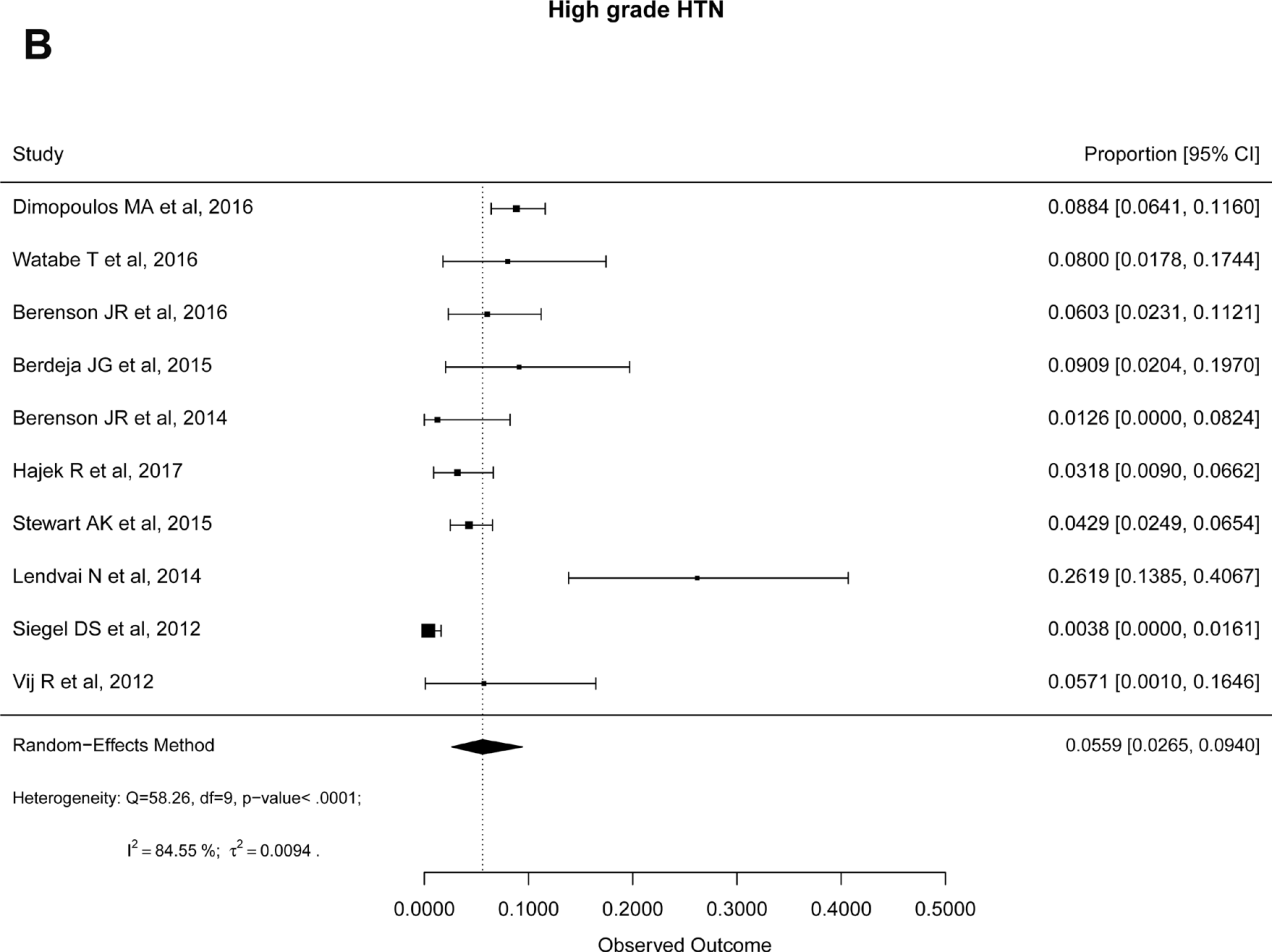
Forest plots of cumulative incidences of high-grade cardiotoxicity (**A**) and hypertension (**B**).

**Table 4:** Response rate analysis: overall and subgroup analysis comparing monotherapy versus combination therapy and high dose versus standard dose of Carf

Response rates	Sub-group	Trials, *N*	Total events, *N*	Total patients, *N*	I^2^ statistics	Response rates % (95% CI)	*P*-value
CR	Overall	12	206	1799	95.79	4.55 (0.61–11.14)	
	**Regimens**						
	Monotherapy	6	6	672	0	0.62 (0.06–1.57)	
	Combination	6	200	1127	94.98	9.74 (2.60–20.32)	0.005
	**Dose of Carf**						
	High	4	73	647	41.68	9.43 (5.81–13.74)	
	Standard	8	133	1152	97.2	3.13 (0.00–13.04)	0.19
VGPR	Overall	13	611	1841	98.05	19.17 (7.44–34.50)	
	**Regimens**						
	Monotherapy	6	47	672	83.9	5.36 (1.45–11.15)	
	Combination	7	564	1169	95.59	36.21 (22.19–51.51)	<0.0001
	**Dose of Carf**						
	High	5	257	689	77.67	30.16 (21.07–40.07)	
	Standard	8	354	1152	98.78	13.83 (0.70–37.68)	0.18
ORR	Overall	14	1088	1887	97.94	45.67 (29.56–62.24)	
	**Regimens**						
	Monotherapy	7	186	718	83.19	24.10 (16.24–32.91)	
	Combination	7	902	1169	89.97	69.66 (59.67–78.8	<0.0001
	**Dose of Carf**						
	High	5	500	689	83.66	65.05 (53.22–76.05)	
	Standard	9	588	1198	98.42	35.70 (15.08–59.47)	0.03
CBR	Overall	14	1240	1887	97.45	56.37 (41.38–70.80)	
	**Regimens**						
	Monotherapy	7	272	718	85.47	35.16 (25.53–45.41)	
	Combination	7	968	1169	86.69	77.92 (69.85–85.07)	0.001
	**Dose of Carf**						
	High	5	545	689	74.74	74.71 (65.75–82.76)	
	Standard	9	695	1198	98.08	38.54 (26.38–51.46)	0.01

Abbreviations: CR, complete response; VGPR, very good partial response; ORR, overall response rate; CBR, clinical benefit rate; Carf, carfilzomib; CI, confidence interval;

Odds ratio (OR) calculated from 3 RCTs showed that Carf significantly improved ORR and CBR rates compared to control groups (OR = 2.4, 95% CI: 1.6–3.4, *P* < 0.0001; OR = 2.02; 95% CI: 1.3–3.03, *P* = 0.0007, respectively). Median time to ORR ranged between 0.95–3.4 months. Median PFS varied from 3.5–8.3 months in studies where Carf was used as a single agent, [17–20, 22, 23] whereas higher median PFS of 5.1–26.3 months was seen in studies in which Carf was used in combination. In the different studies, median OS in Carf groups varied from 10–47.6 months, however, OS was not usually chosen as a primary endpoint [12–25].

### Response rates –subgroup analyses

As shown in Table 4, combination regimen compared to monotherapy showed significantly better response rates. A total of 5 studies used higher (>20/27 mg/m^2^/day) dose of Carf and 4 out of these 5 studies used it in a combination regimen. High dose of Carf showed better CR and VGPR rates when compared to standard dose Carf but was not statistically significant (*P* ≥ 0.18). However, ORR was significantly higher with a high dose of Carf at 65% (95% CI: 53–76) compared to 35% using standard dose (95% CI: 15–59) with *P* = 0.03. Similarly, high dose of Carf significantly (*P* = 0.01) improved CBR to 74% (95% CI: 65–82) over that of 38% (95 % CI: 26–51) from standard dose. We found no significant difference in ORR between patients with high risk and standard risk cytogenetics treated by Carf based regimens (OR 0.84, 95% CI: 0.59–1.18, *P* = 0.62). Similarly, no significant (*P* ≥ 0.32) difference in ORR was found between the different ISS stages (33%, 22%, 27%, respectively for ISS stage I, II, and III).

### Common adverse events

Common high-grade (grade ≥ 3) hematological adverse events reported were anemia (15.3%), thrombocytopenia (17%), neutropenia (15%), and lymphopenia (23%). Similarly, cumulative incidences of high-grade non-hematological adverse events reported were pneumonia (8%), fatigue (6%), cardiotoxicity (5%) (Figure 2A), HTN (5%) (Figure 2B), renal toxicity (5%), diarrhea (2%), upper respiratory airways infection (1%), pyrexia (1%), nausea (1%) and peripheral neuropathy (1%). When compared to control group from 3 RCTs, we found that the odds of developing cardiotoxicity and HTN were significantly higher in Carf group with OR 2.04 (95% CI: 1.31–3.17, *P* = 0.002) and 3.33 (95% CI: 1.98–5.60, *P* < 0.0001), respectively (Table 5).

**Figure 3:**
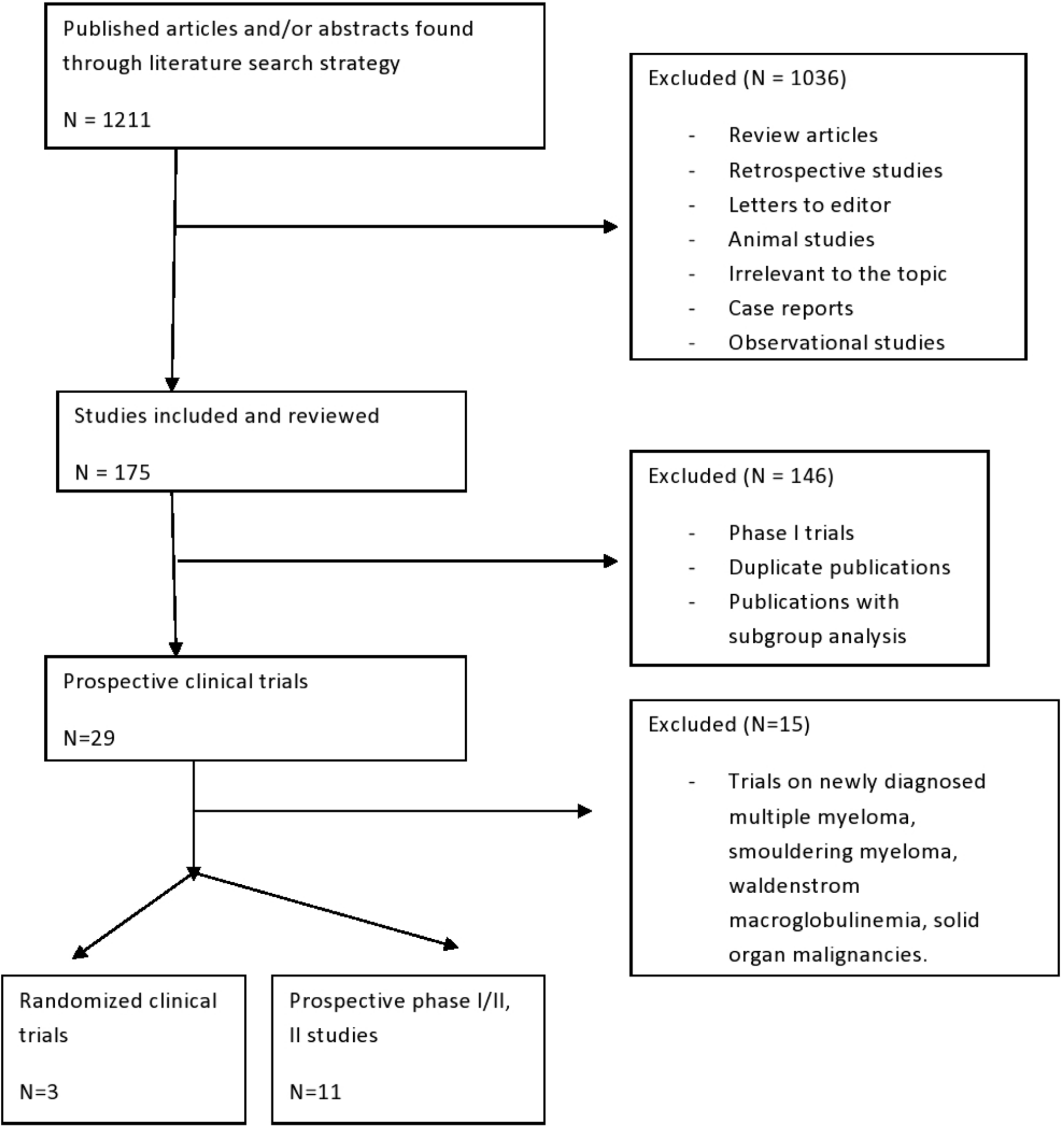
Schema of step by step process for selection of studies included in this meta-analysis

**Table 5:** Odds ratio (OR) calculations for common adverse events comparing events in Carf versus control groups from phase III trials

Adverse events	No. of trials	Total events, *N*	Total pts, *N*	I^2^ statistics	OR (95% CI)	*P*-value
**Hematological**						
Anemia	3	336	2036	55.78	1.12 (0.78–1.62)	0.53
Thrombocytopenia	3	267	2036	8.72	1.16 (0.88–1.53)	0.28
Neutropenia	2	250	1107	60.47	0.93 (0.50–1.74)	0.81
**Non-hematological**						
Neuropathy	3	70	2036	65.46	0.54 (0.18–1.65)	0.28
Renal toxicity	3	90	2036	56.46	1.85 (0.93–3.67)	0.07
Fatigue	2	112	1721	25.82	0.97 (0.62–1.51)	0.87
Diarrhea	2	80	1721	51.76	0.64 (0.33–1.27)	0.20
Nausea	2	13	1244	0	1.60 (0.51–4.99)	0.41
Upper respiratory infection	2	23	1721	0	2.28 (0.93–5.61)	0.07
Pyrexia	3	28	2036	0	4.13 (1.61–10.58)	0.001
Pneumonia	1	29	315	0	0.50 (0.22–1.11)	0.08
Cardiotoxicity	3	61	2036	0	2.04 (1.31–3.17)	0.002
Hypertension	3	64	2036	0	3.33 (1.98–5.60)	<0.0001

Abbreviations: OR, odds ratio; CI, confidence interval.

### Common adverse events –subgroup analyses

We performed analyses based on regimen used (monotherapy vs. combination) and a dose of Carf (high dose vs. standard dose) for all common high-grade adverse events mentioned above. Interestingly, the incidences of all commonly reported high-grade toxicities including cardiotoxicity were not significantly different between high versus standard Carf doses except for HTN (Supplementary Table 1). A trend towards a higher cumulative incidence of HTN was found at 8.7% with high dose Carf (95% CI: 4–14.6%) as compared to 3.06% with standard dose (95% CI: 0.7–6.5%). Moreover, the incidences of all commonly reported high-grade toxicities were not significantly different between combination therapy and monotherapy except for peripheral neuropathy (1.6% vs. 0.3%, respectively; *P* = 0.04) and diarrhea (3.3% vs. 0.5%, respectively; *P* = 0.002) (Supplementary Table 1). Cumulative incidences of cardiotoxicity were not significantly different between high dose vs. standard dose of Carf (5.7% vs. 4.9%) and combination vs. monotherapy (6.2% vs. 4%) of Carf (Supplementary Table 1).

### Bias

No publication bias was detected by visual inspection of funnel plots and by Egger's tests. Study quality and risks of biases were assessed using the Cochrane Collaboration's tools. Among the RCTs, the risk of selection bias and attrition bias were low while performance bias, detection bias, and reporting bias were unclear as per Cochrane Collaboration's tools. Among non-randomized trials, the overall risks of biases were low.

## DISCUSSION

To our knowledge, this is the first meta-analysis incorporating 14 clinical trials which used Carf based regimens, analyzing data on 2906 RRMM patients.

By analyzing the published data, we found the ORR and CBR to be 45% and 56%, respectively, with Carf based regimens. Median PFS ranged from 3.7–18.7 months, and median OS ranged from 10.2–47.6 months [12–27]. Prognosis in MM is a highly complex issue as PFS and OS can be influenced by multiple diseases and patient-related factors. The ASPIRE and ENDEAVOR studies demonstrated significantly improved median PFS and OS [26, 27]. On the other hand, the FOCUS study did not show the difference in the median PFS (*P* = 0.24) and median OS (*P* = 0.41) for the Carf group when compared with control group [17]. Multiple factors could have played a role in this reported wide variability in response benefit; one of them could be differences in enrollment criteria such as the FOCUS study enrolled patients who had received median of 5 previous treatment lines (ENDEAVOR and ASPIRE had median of 2 previous treatment lines), more patients with ECOG status ≥ 2 (19%) in FOCUS trial (ENDEAVOR and ASPIRE did not have any patients with ECOG >2), single-agent Carf use in FOCUS trial, and also higher percentage of patients with PD (17%) at the time of enrollment in the FOCUS trial. Regardless, Carf seems to offer much better OS as a median OS of 9 months is typically seen for patients who are refractory to Bort and/or IMiDs [6]. The responses to Carf appeared to be durable, with median DOR ranging between 7.2–28.6 months, lower with single-agent [17–23] while higher with combination therapy.

ASPIRE study excluded patients who progressed on Bort, showed the highest ORR (87%) and CBR (91%) to Carf. Vij *et al.* [22, 23] showed patients who were Bort naïve had better ORR (47%) and CBR (61%) compared to patients refractory to Bort {ORR (17%) and CBR (31%)}. Collectively, this information suggests that while Carf is efficacious in patients previously treated with Bort, the response rates were not as robust. The mechanism by which Carf overcomes resistance to Bort is unclear [28], but it may partially be due to the irreversible nature of proteasome inhibition with more selective inhibition by Carf [28–30]. As shown in results, ORR (*P* = 0.03) and CBR (*P* = 0.01) were significantly better with higher dose Carf (>20/27 mg/m^2^/day) compared to standard Carf dose, irrespective of regimen (monotherapy or combination). This is consistent with the results of preclinical studies [28–30]. Moreover, preclinical data [31] suggested that a slower infusion over a longer period (30 min infusion as opposed to 10 min) compared to a rapid infusion of the same dose of Carf resulted in a better tolerance of Carf with the potential for greater and more prolonged proteasome inhibition and improved efficacy. As shown in results, while high dose of Carf offered better response rates, it was without any significantly added toxicities except for HTN (*P* = 0.05). While the most frequently used higher Carf dose was 56 mg/m^2^/day, the most effective and safe higher Carf dose remains to be determined.

Most high-risk disease factors including unfavorable cytogenetics and higher ISS stages are associated with short-lived remissions, rapid relapses and aggressive disease [32]. Our analysis shows no difference in ORR between patients with standard-risk versus high-risk cytogenetics (*P* = 0.62) among those treated by Carf. Furthermore, our analysis also shows that Carf is equally efficacious for all ISS stages. These findings suggest that Carf could be used in wide spectrum of patient populations including patients with unfavorable cytogenetic abnormalities and advanced ISS stage. However, Carf does not seem to completely overcome the overall poor prognosis of high risk patients who show lower PFS and OS compared to standard risk patients [33].

Our analysis shows that the most commonly reported adverse events were hematological with no significant difference between Carf and control group. Interestingly, ORs for developing cardiotoxicity (*P* = 0.002) and HTN (*P* < 0.0001) were significantly higher with Carf [43]. Cardiotoxicity of these novel agents is thought to be the result of modulation of endothelial nitric oxide synthase (eNOS) activity and nitric oxide (NO) levels by proteasome inhibition [34]. Thus, Carf being irreversible PI would provide a prolonged inhibition which could result in sustained oxidative stress in some patients and lead to higher cardiotoxicity incidence. On the other hand, higher incidence of HTN induced by Carf was suggested to be due to an autonomic neuropathy induced by proteasome inhibition [25, 35]. It is recommended that patients receiving Carf be closely monitored for cardiac complications, however, proper monitoring strategy needs to be determined. Moreover, identifying predisposing factors for cardiotoxicity needs further research as traditional cardiovascular risk factors did not show any association in a retrospective analysis [36].

Our meta-analysis shows that the cumulative incidence of high-grade (≥3) peripheral neuropathy reported in Carf trials was 1.1%, which seems to be much lower than the 8.1% reported in Bort trials [37]. Indeed peripheral neuropathy is a major dose-limiting side effect with Bort treatment [38, 39] and published reports suggest the possibility of underlying genetic factors for the development of Bort induced peripheral neuropathy [40]. Other explanations for less neuropathy with Carf could be due to being more selective [41] and faster clearance from the circulation after intravenous administration [31]. Furthermore, studies have also found that baseline peripheral neuropathy does not impact the efficacy and tolerability of Carf [42]. All these observations point to an advantage of using Carf in patients who have already had existing neuropathy from prior exposure to Bort, knowing that it may not get worse.

As with all other meta-analyses, ours has a few limitations: 1) This analysis was based on the published data of clinical trials, whereas an individual level data-based analysis would have more detailed information on patient variables; 2) Patients enrolled in trials usually have adequate organ function and are relatively healthier compared to the patients in common oncology practice; 3) The reporting of cardiotoxicity was highly variable among different studies, [14–16, 21, 25] where some studies reported it in a broad category as “cardiac failure”, while others used more specific terminology such as congestive heart failure, arrhythmias, atrioventricular block, cardiomyopathy or cardiac arrest. Dyspnea was used as the pulmonary adverse event in earlier studies, although it is likely resulting from pulmonary congestion caused by congestive heart failure [20, 25]; 4) Marked heterogeneity between studies. We chose the random-effects model for all calculations to increase power and precision; 5) Majority of studies which used a high dose of Carf, used variable doses and due to lack of individual patient level data, we were not able to perform analysis to see which higher dose, in particular, is most effective and safest.

In conclusion, our analysis shows treatment with Carf based regimens offers significantly better response rates and survival rates with an acceptable safety profile in patients with RRMM. Combination regimens compared to single-agent Carf and high versus standard dose seem to offer better response rates with an acceptable toxicity profile. Moreover, Carf seems to be efficacious irrespective of the cytogenetics and ISS stage. The cumulative incidences of cardiotoxicity and HTN are higher in patients treated with Carf and the odds of developing HTN increases with the use of higher Carf dose. Finally, the incidence of peripheral neuropathy, unlike with Bort, does not seem to be an issue with Carf.

## MATERIALS AND METHODS

The selection and systematic review of trials were performed in accordance with the Preferred Reporting Items for Systematic Reviews and Meta-Analysis (PRISMA) statement [44].

### Literature search strategy

Two investigators (CS and RB) conducted an independent literature search of PubMed, Web of Science, and clinical trial registry (http://clinicaltrials.gov). We also searched abstracts from American Society of Clinical Oncology and American Society of Hematology conferences that took place up until January 2017. Key words used were carfilzomib, Kyprolis, PR-171, and cancer. Reference list of selected studies and other published systematic reviews were also searched separately to capture any relevant studies. Studies with full article published prior to January 2017 were selected. In the case of multiple publications originating from a single trial or duplicate publications, only the most recent and updated report of the clinical trial was included.

### Inclusion and exclusion criteria

All the studies enrolled patients who relapsed after receiving ≥ 1 previous lines of therapy which usually included Bort, Len and/or Thal. Vij *et al.* [23] enrolled and studied Bort naïve patients separately. ASPIRE study excluded patients who progressed during treatment with Bort [21]. Berenson *et al.* enrolled only those patients who relapsed within 12 weeks of receiving or were refractory to their most recent Bort-containing regimen [15].

### Selection of studies and data extraction

The primary goal of the meta-analysis was to analyze response rates of Carf in RRMM patients and carry out sub-group analyses. Secondary goals were to analyze common adverse events reported in trials as well as perform analyses to assess the effects of reported disease variables on responses and outcomes. We included only prospective trials published prior to January 2017 and written in the English language. Studies were included if the participants were assigned to treatment with Carf as a single agent or in combination with other agents. We excluded a total of 32 phase I studies as our primary goal is to analyze efficacy of Carf. Complete step by step selection process of clinical trials is described in Figure 3.

Two investigators (CS and RB) independently conducted the data extraction from 14 included studies, and any discrepancy between the two was resolved by consensus. These data include first author's name, year of publication, phase of clinical trials, underlying malignancy and histology, disease stage and disease characteristics, inclusion and exclusion criteria, total number of enrolled patients and controls, the median age of patients, dose of Carf, treatment regimen, response categories as per International Myeloma Working Group Uniform response criteria [45] such as CR,VGPR, partial response (PR), ORR, stable disease (SD), progressive disease (PD), and common adverse events. Furthermore, minimal response (MR) and CBR were also gathered as mentioned in the respective studies as per European Blood and Marrow Transplantation Group criteria [46]. When reported, we also gathered the following data: PFS, OS, the median duration of treatment, median time to overall response, the median duration of overall response. As for the adverse events, studies recorded the adverse events as all-grade or high-grade based on the Common Terminology Criteria for Adverse Events (CTCAE) version 2, 3, or 4, which is widely accepted in clinical trials [47]. Adverse events were not included in calculations whenever it was specifically reported in the article that the events were not secondary to the drug of interest. Adverse cardiac events reported were: acute coronary syndrome, acute left or right ventricular failure, acute myocardial infarction, angina pectoris, various arrhythmias, cardiac arrest, cardiac failure and cardiomyopathy. Details of reported cardiac events from individual studies are mentioned in Tables 1, 2, and 3.

### Subgroup analyses

First, in order to analyze the difference in the response rates and adverse event rates based on the dose of Carf used, we divided studies into two groups: studies that used standard dose (≤ 27 mg/m^2^) and those used high dose (such as 36, 45, 56, 70, 88 mg/m^2^). In a second analysis, we calculated the response rates and adverse event rates based on the regimen used, such as Carf monotherapy versus combination with other agents. Furthermore, we also analyzed the effect of high risk cytogenetics [such as t(4;14), t(14;16), or deletion 17p] and disease status based on ISS stage on the response rates and outcomes of patients treated with Carf based regimens.

### Study quality and statistical analysis

Study quality and risk of bias was assessed using the Cochrane Collaboration's tools [48]. The pooled cumulative incidences of toxicities and its 95% confidence intervals (CI) were derived by random-effects model (DerSimonian-Laird estimator) based on the reported number of patients with events of interest among evaluable patients that received Carf in respective studies. From studies with control groups, pooled OR and its 95% CIs were also calculated by random-effects model (DerSimonian-Laird estimator). In subgroup analyses, Satterthwaite *T*-test was applied to compare the two proportions. All tests with *P*-value < 0.05 were considered statistically significant. The heterogeneity between studies was assessed by I-squared (I^2^) statistic, where values <25%, 25–50% and >50% were considered as a low, moderate and high degree of heterogeneity, respectively [49]. The publication bias was assessed by visual inspection of the funnel plots and Egger's tests [50]. All statistics were calculated using “ R “ software with metafor package [51].

## SUPPLEMENTARY MATERIALS FIGURES AND TABLES





## Abbreviations

MM: Multiple myeloma
PI: Proteasome inhibitor
IMiDs: Immunomodulators
Bort: Bortezomib
Carf: Carfilzomib
Dexa: Dexamethason
Len: Lenalidomide
RRMM: relapsed and/or refractory multiple myeloma
DOR: Duration of response
PFS: Progression free survival
OS: overall survival
CR: Complete response
VGPR: Very good partial response
PD: progressive disease
MR: Minimal response
ORR: Overall response rate
CBR: Clinical benefit rate
SD: Stable disease
ISS: International staging system
OR: Odds ratio
HTN: Hypertension
