# Supplementary material for: Efficacy and safety of carfilzomib in relapsed and/or refractory multiple myeloma: systematic review and meta-analysis of 14 trials
Source: Oncotarget. 2018 May 4;9(34):23704–17. doi: 10.18632/oncotarget.25281 (PMC5955098; doi:10.18632/oncotarget.25281)
Supplement: Supplementary file 1 [file oncotarget-09-23704-s001.pdf]

Efficacy and safety of carfilzomib in relapsed and/or refractory multiple myeloma: systematic review and meta-analysis of 14 trials

SUPPLEMENTARY MATERIALS

Supplementary Table 1: Cumulative incidence of common high-grade adverse events with subgroup analysis based on Carf dose and type of regimen based on selected clinical trials done in RRMM patients. See Supplementary_Table_1
