# Supplementary material for: Efficacy and safety of carfilzomib in relapsed and/or refractory multiple myeloma: systematic review and meta-analysis of 14 trials
Source: Oncotarget. 2018 May 4;9(34):23704–17. doi: 10.18632/oncotarget.25281 (PMC5955098; doi:10.18632/oncotarget.25281)
Supplement: Supplementary file 2 [file oncotarget-09-23704-s002.docx]

| **Supplementary Table 1: Cumulative incidence of common high-grade adverse events with subgroup analysis based on Carf dose and type of regimen based on selected clinical trials done in RRMM patients.** | | | | | | | |
| --- | --- | --- | --- | --- | --- | --- | --- |
| **Adverse events. High grade** | **Subgroups** | **Trials, n** | **Events, n** | **Patient, n** | **I^2^ Statistics(%)** | **Overall effect %**  **(95%CI)** | **P value** |
|  | | | | | | | |
| **Anemia** | **Overall** | 13 | 270 | 1651 | 77.16 | 15.3(11.37-19.66) |  |
|  | **Regimens** |  | | | | | |
|  | Combination | 7 | 175 | 1184 | 65.54 | 13.35(9.59-17.59) | 0.34 |
|  | Monotherapy | 6 | 95 | 467 | 82.88 | 17.88(9.76-27.7) |  |
|  | **Carf Dose** |  | | | | | |
|  | high | 5 | 89 | 704 | 62.70 | 11(6.43-16.51) | 0.07 |
|  | standard | 8 | 181 | 947 | 76.48 | 18.12(12.72-24.19) |  |
|  | | | | | | | |
| **Thrombocytopenia** | **Overall** | 13 | 256 | 1651 | 86.56 | 17.64(12.38-23.57) |  |
|  | **Regimens** |  | | | | | |
|  | Combination | 7 | 171 | 1184 | 90.03 | 18.95(11.46-27.74) | 0.64 |
|  | Monotherapy | 6 | 85 | 467 | 80.19 | 16.22(8.94-25.06) |  |
|  | **Carf Dose** |  | | | | | |
|  | high | 5 | 85 | 704 | 91.37 | 18.99(8.05-32.97) | 0.80 |
|  | standard | 8 | 171 | 947 | 75.47 | 17.35(12.16-23.22) |  |
|  | | | | | | | |
| **Neutropenia** | **Overall** | 11 | 225 | 1137 | 91.66 | 15.7(8.52-24.47) |  |
|  | **Regimens** |  | | | | | |
|  | Combination | 6 | 173 | 720 | 92.24 | 18.89(8.42-32.18) | 0.39 |
|  | Monotherapy | 5 | 52 | 417 | 87.36 | 12.12(4.16-23.16) |  |
|  | **Carf Dose** |  | | | | | |
|  | high | 4 | 25 | 240 | 79.87 | 12(3.8-23.55) | 0.44 |
|  | standard | 7 | 200 | 897 | 93.02 | 17.76(8.49-29.37) |  |
| **Peripheral neuropathy** | **Overall** | 11 | 25 | 1707 | 0.00 | 1.12(0.58-1.79) |  |
|  | **Regimens** |  | | | | | |
|  | Combination | 7 | 22 | 1184 | 0.00 | 1.6(0.86-2.51) | 0.04 |
|  | Monotherapy | 4 | 3 | 523 | 0.00 | 0.31(0-1.24) |  |
|  | **Carf Dose** |  | | | | | |
|  | high | 5 | 11 | 704 | 0.00 | 1.33(0.47-2.49) | 0.44 |
|  | standard | 6 | 14 | 1003 | 17.60 | 0.94(0.25-1.93) |  |
|  | | | | | | | |
| **Renal toxicity** | **Overall** | 10 | 90 | 1625 | 74.44 | 5.62(3.24-8.53) |  |
|  | **Regimens** |  | | | | | |
|  | Combination | 5 | 39 | 1056 | 0.00 | 3.29(2.21-4.55) | 0.03 |
|  | Monotherapy | 5 | 51 | 569 | 78.98 | 8.94(3.87-15.64) |  |
|  | **Carf Dose** |  | | | | | |
|  | high | 4 | 26 | 660 | 0.00 | 3.5(2.1-5.18) | 0.09 |
|  | standard | 6 | 64 | 965 | 83.84 | 7.51(3.36-12.98) |  |
|  | | | | | | | |
| **Fatigue** | **Overall** | 12 | 119 | 1710 | 2.85 | 6.54(5.32-7.87) |  |
|  | **Regimens** |  | | | | | |
|  | Combination | 7 | 79 | 1184 | 13.04 | 6.33(4.74-8.1) | 0.52 |
|  | Monotherapy | 5 | 40 | 526 | 0.00 | 7.2(5.04-9.68) |  |
|  | **Carf Dose** |  | | | | | |
|  | high | 5 | 43 | 704 | 33.74 | 5.99(3.44-9.09) | 0.52 |
|  | standard | 7 | 76 | 1006 | 0.00 | 7.2(5.61-8.95) |  |
|  | | | | | | | |
| **Diarrhea** | **Overall** | 9 | 45 | 1489 | 36.70 | 2.58(1.44-3.99) |  |
|  | **Regimens** |  | | | | | |
|  | Combination | 6 | 43 | 1142 | 0.00 | 3.36(2.31-4.58) | 0.002 |
|  | Monotherapy | 3 | 2 | 347 | 0.00 | 0.55(0-1.9) |  |
|  | **Carf Dose** |  | | | | | |
|  | high | 4 | 23 | 662 | 0.00 | 3.02(1.71-4.61) | 0.48 |
|  | standard | 5 | 22 | 827 | 59.19 | 2.22(0.57-4.66) |  |
|  | | | | | | | |
| **Nausea** | Overall | 8 | 16 | 1134 | 0.00 | 1.14(0.49-1.97) |  |
|  | **Regimens** |  | | | | | |
|  | Combination | 4 | 8 | 630 | 0.00 | 1(0.24-2.11) | 0.61 |
|  | Monotherapy | 4 | 8 | 504 | 0.00 | 1.33(0.36-2.72) |  |
|  | **Carf Dose** |  | | | | | |
|  | high | 3 | 8 | 546 | 15.44 | 1.2(0.1-3.07) | 0.94 |
|  | standard | 5 | 8 | 588 | 0.00 | 1.26(0.38-2.49) |  |
|  | | | | | | | |
| **Upper respiratory tract infection** | **Overall** | 9 | 34 | 1495 | 0.00 | 1.83(1.12-2.68) |  |
|  | **Regimens** |  | | | | | |
|  | Combination | 5 | 19 | 1098 | 0.00 | 1.35(0.64-2.25) | 0.02 |
|  | Monotherapy | 4 | 15 | 397 | 0.00 | 3.54(1.79-5.75) |  |
|  | **Carf Dose** |  | | | | | |
|  | high | 3 | 10 | 618 | 0.00 | 1.18(0.33-2.38) | 0.19 |
|  | standard | 6 | 24 | 877 | 11.48 | 2.4(1.27-3.81) |  |
|  | | | | | | | |
| **Pyrexia** | **Overall** | 7 | 29 | 1448 | 0.00 | 1.58(0.9-2.4) |  |
|  | **Regimens** |  | | | | | |
|  | Combination | 3 | 18 | 944 | 0.00 | 1.46(0.68-2.46) | 0.59 |
|  | Monotherapy | 4 | 11 | 504 | 0.00 | 1.84(0.69-3.38) |  |
|  | **Carf Dose** |  | | | | | |
|  | high | 1 | 11 | 464 | - | - | - |
|  | standard | 6 | 18 | 984 | 0.00 | 1.52(0.73-2.50) |  |
|  | | | | | | | |
| **Pneumonia** | **Overall** | 11 | 88 | 1019 | 33.80 | 8.19(6.03-10.61) |  |
|  | **Regimens** |  | | | | | |
|  | Combination | 4 | 22 | 286 | 55.20 | 7.84(3.5-13.54) | 0.80 |
|  | Monotherapy | 7 | 66 | 733 | 24.17 | 8.51(6.1-11.25) |  |
|  | **Carf Dose** |  | | | | | |
|  | high | 3 | 16 | 202 | 70.12 | 8.32(2.11-17.58) | 0.99 |
|  | standard | 8 | 72 | 817 | 14.00 | 8.42(6.33-10.75) |  |
|  | | | | | | | |
| **Cardiotoxicity** | **Overall** | 13 | 111 | 1882 | 26.83 | 5.25(3.96-6.7) |  |
|  | **Regimens** |  | | | | | |
|  | Combination | 7 | 78 | 1184 | 0.00 | 6.19(4.82-7.71) | 0.17 |
|  | Monotherapy | 6 | 33 | 698 | 50.24 | 4.09(1.95-6.83) |  |
|  | **Carf Dose** |  | | | | | |
|  | high | 5 | 44 | 704 | 0.00 | 5.76(4.05-7.72) | 0.48 |
|  | standard | 8 | 67 | 1178 | 46.99 | 4.92(3.11-7.06) |  |
|  | | | | | | | |
| **Hypertension** | **Overall** | 10 | 92 | 1608 | 84.55 | 5.59(2.65-9.4) |  |
|  | **Regimens** |  | | | | | |
|  | Combination | 6 | 80 | 1100 | 78.18 | 7.52(3.87-12.13) | 0.14 |
|  | Monotherapy | 4 | 12 | 508 | 77.78 | 2.94(0.18-7.91) |  |
|  | **Carf Dose** |  | | | | | |
|  | high | 5 | 63 | 704 | 72.33 | 8.7(4.09-14.65) | 0.05 |
|  | standard | 5 | 29 | 904 | 77.10 | 3.06(0.75-6.54) |  |

Abbreviations: CI, Confidence interval; Carf, Carfilzomib.
